# Association of isolated minor nonspecific ST-T abnormalities with left ventricular hypertrophy and diastolic dysfunction

**DOI:** 10.1038/s41598-018-27028-6

**Published:** 2018-06-08

**Authors:** Jeong Gyu Kang, Yoosoo Chang, Ki-Chul Sung, Jang-Young Kim, Hocheol Shin, Seungho Ryu

**Affiliations:** 1 Center for Cohort Studies, Total Healthcare Center, Kangbuk Samsung Hospital, Sungkyunkwan University, School of Medicine, Seoul, South Korea; 2 Department of Occupational and Environmental Medicine, Kangbuk Samsung Hospital, Sungkyunkwan University, School of Medicine, Seoul, South Korea; 3 Department of Clinical Research Design & Evaluation, SAIHST, Sungkyunkwan University, Seoul, South Korea; 4 Division of Cardiology, Department of Internal Medicine, Kangbuk Samsung Hospital, Sungkyunkwan University School of Medicine, Seoul, South Korea; 5 Departments of Cardiology, Wonju College of Medicine, Yonsei University, Wonju, South Korea; 6 Institute of Genomic Cohort, Wonju College of Medicine, Yonsei University, Wonju, South Korea; 7 Department of Family Medicine, Kangbuk Samsung Hospital, Sungkyunkwan University School of Medicine, Seoul, South Korea

## Abstract

The aim of this study was to examine the associations of isolated minor nonspecific ST-T abnormalities (NSSTTA) on 12-lead electrocardiogram (ECG) with left ventricular (LV) diastolic function and LV geometry on echocardiography. A cross-sectional study comprised of 74,976 Koreans who underwent ECG and echocardiography as part of a comprehensive health examination between March 2011 and December 2014. ECG was coded using Minnesota Code criteria. The frequencies of NSSTTA, impaired LV relaxation, and echocardiographic LVH were 1,139 (1.5%), 21,118 (28.2%), and 1,687 (2.3%) patients, respectively. The presence of NSSTTA was positively associated with the prevalence of impaired LV relaxation and LVH on echocardiography. In a multivariable-adjusted model, the odds ratio (95% CIs) comparing patients with NSSTTA to control patients was 1.55 (1.33–1.80) for impaired LV relaxation and 3.15 (2.51–3.96) for echocardiographic LVH. The association between NSSTTA and impaired LV relaxation was stronger in the intermediate to high cardiovascular disease-risk group than in the low-risk group according to Framingham Risk Score stratification (P for interaction = 0.02). NSSTTA were associated with increased prevalence of impaired LV relaxation and LVH, suggesting NSSTTA as an early indicator of subclinical cardiac dysfunction and geometric abnormalities.

## Introduction

Heart failure is a progressive disease associated with aging, and up to half of heart failure cases are attributed to diastolic dysfunction^1^. Decreased early mitral annulus velocity, a measure of impaired left ventricular (LV) relaxation, was associated with fatal and nonfatal cardiovascular events, including overt heart failure^2,3^. LV hypertrophy (LVH) is a strong predictor of non-fatal and fatal cardiovascular events^4^. Therefore, it is important to identify impaired LV relaxation and LVH in asymptomatic individuals for establishing preventive strategies before adverse cardiovascular events occur.

Electrocardiogram (ECG) is an inexpensive and convenient tool to assess the geometric and functional status of the heart and is widely used in clinical practice. Furthermore, ECG can reveal past heart events and predict future cardiovascular disease (CVD). Isolated minor nonspecific ST-T wave abnormalities (NSSTTA), one of the most common ECG abnormalities, are considered a benign finding in asymptomatic individuals, but several studies have found that NSSTTA are associated with increased risk of cardiovascular events or death^5–7^. However, the precise mechanism underlying the poor cardiovascular prognosis of NSSTTA has not been fully elucidated, and the echocardiographic characteristics of NSSTTA have not been explored yet.

Therefore, the goal of this study was to examine the associations between NSSTTA and echocardiographic findings, including geometric changes and LV functional status, in a large sample of Korean men and women who participated in a health check-up program.

## Methods

### Study population

The Kangbuk Samsung Health Study is a cohort of Korean men and women who underwent comprehensive annual or biennial examinations at Kangbuk Samsung Hospital Total Healthcare Centers in Seoul and Suwon, South Korea^8,9^. Over 80% of the participants were employees of various companies or local government organizations and their spouses, and the health screening exams were paid for by employers under the Korean Industrial Safety and Health Law. The remaining participants voluntarily purchased self-paid screening exams at the health screening center. This study consisted of 74,976 men and women who underwent echocardiography as part of a comprehensive health examination between March 2011 and December 2014.

We excluded 11,741 participants for the following reasons: missing data on either ECG, levels of serum glucose, low-density lipoprotein cholesterol (LDL-C), high-density lipoprotein cholesterol (HDL-C), or triglycerides, body mass index (BMI), or systolic blood pressure (BP) (n = 393); a history of malignancy (n = 2176); a history of CVD (n = 1085); decreased LV systolic function (ejection fraction <50%), hypertrophic, dilated and ischemic cardiomyopathy, mitral/aortic stenosis of mild grade or greater, moderate or higher grade of mitral/aortic regurgitation, or post-operative cardiac surgery including valve replacement (n = 3213); or the presence of major ECG abnormalities according to the Minnesota Code (n = 4874). Because some participants met more than one exclusion criteria, 74,976 participants were included in this study. This study was approved by the Institutional Review Board of Kangbuk Samsung Hospital, and the requirement for informed consent was waived because we used de-identified retrospective data that had been routinely collected during the health screening process. We confrm that all methods were performed in accordance with the relevant guidelines and regulations.

### Measurements

Information on demographic characteristics, smoking status, alcohol consumption, education level, and medical history were collected by standardized, self-administered questionnaires as previously described^8^. Smoking status categories included never, former, and current. Alcohol consumption was categorized into none, moderate (≤20 g/day), and high (>20 g/day). The physical activity was assessed using the validated Korean version of the International Physical Activity Questionnaire Short Form^10^. Health-enhancing physically active (HEPA) was defined as physical activity that meets either of two criteria: (i) vigorous intensity activity on three or more days per week accumulating ≥1500 MET min/week; or (ii) seven days of any combination of walking, moderate intensity, or vigorous intensity activities achieving at least 3000 MET min/week^10^.

Height and weight were measured by trained nurses and BMI was calculated as height (m) divided by weight (kg) squared (m/kg^2^). Obesity was defined as BMI ≥ 25 m/kg^2^ according to the proposed criteria for obesity in Asian populations. BP was measured using an automated oscillometric device (Model 53000; Welch Allyn, New York, USA) while subjects were in a sitting position with the arm supported at heart level. Three readings were recorded for each individual, and the average BP of the second and third readings was used for analysis to reduce errors in measurement. Hypertension was defined as a systolic BP ≥ 140 mmHg, a diastolic BP ≥ 90 mmHg, or current use of antihypertensive medication.

Measurements for serum biochemical parameters, including levels of glucose, insulin, high sensitivity C-reactive protein (hsCRP), total cholesterol, triglycerides, LDL-C, and HDL-C, are described in detail elsewhere^8^. Insulin resistance was assessed with the homeostatic model assessment – insulin resistance (HOMA-IR) equation: fasting blood insulin (uU/mL) × fasting blood glucose (mmol/L)/22.5. Diabetes was defined as a fasting serum glucose ≥126 mg/dL, hemoglobin A1c ≥ 6.5%, or current use of insulin or anti-diabetic medications.

### 12-lead electrocardiogram

Standard 12-lead ECG were obtained at a 25 mm/s paper speed and at 1 mV/cm amplification with an ECG recorder (CARDIMAX FX-7542; Fukuda Denishi Co., Ltd., Tokyo, Japan). ECG was performed in all subjects the same day as the echocardiographic examination and was interpreted by two experienced cardiologists who were blinded to the echocardiographic results and codified with the Minnesota Code^11^. Criteria for major ECG abnormalities were any of the following^5^: Q-QS wave abnormalities, major ST-T wave abnormalities, LVH, atrial fibrillation, atrial flutter, Wolff-Parkinson-White syndrome, complete bundle-branch block, and intraventricular block. NSSTTA were classified according to Minnesota Codes 4-3, 4-4, 5-3, and 5-4.

### Echocardiography

Conventional echocardiography was performed with ultrasound scanners (Vivid 7 and E9; General Electric, Milwaukee, WI, USA) by registered diagnostic cardiac sonographers. Linear measurements of LV posterior wall thickness (PWT), intraventricular septum thickness (IVST), and diameter of the LV cavity at the end of diastole and systole were obtained in M-mode in the parasternal long axis view. The LV ejection fraction was calculated from LV end-diastolic diameter (LVEDD) and end-systolic diameter (LVESD) using the Teichholz method. The LV mass was calculated with measurements obtained in M-mode using the following equation: LV mass = 0.8 × [1.04 × (LVEDD + IVST + PWT)^3^ − LVESD^3^] + 0.6 g^12^. The LV mass index (LVMI) was calculated as LV mass/height^2.7^, and LVH was defined as LVMI ≥ 45 g/m^2.7^ for women and ≥49 g/m^2.7^ for men^13^. The anteroposterior dimension of the left atrium (LA) was also measured.

To assess diastolic function, the pulse-wave Doppler transmitral LV inflow velocity in the apical 4-chamber view was sampled. The early diastolic mitral inflow peak velocity (E), late diastolic peak velocity (A) during atrial contraction, and deceleration time of the E velocity (DT) were measured. The early (E′) and late (A′) velocities from tissue Doppler imaging of the septal mitral annulus were also measured. Impaired LV relaxation was defined as E′ < 8 cm/s^14,15^.

### Statistical analyses

Descriptive statistics were used to summarize the characteristics of participants by the presence of NSSTTA. Age- and sex-adjusted mean values and 95% CIs of echocardiographic findings were also examined according to the presence of NSSTTA.

To determine the association of NSSTTA with impaired LV relaxation and LVH, we used a logistic regression model to estimate odds ratios with 95% confidence intervals (CIs). We used three models with progressively increased adjustment for confounding variables. Data were initially adjusted for age and sex and then were further adjusted for study center (Seoul or Suwon), year of screening exam, smoking history (never, past, current, or unknown), alcohol intake (0, <20, ≥20 g/d, or unknown), physical activity, educational level (high school graduate or less, community college or university graduate, graduate school or higher, and unknown), and BMI. Finally, model 2 was further adjusted for family history of heart disease; history of diabetes and history of hypertension; levels of glucose, LDL-cholesterol, HDL-cholesterol, and triglycerides; and systolic BP.

We performed stratified analyses in pre-specified subgroups defined by sex (male vs. female), age (<50 vs. ≥50 years), smoking (never or ex-smoker vs. current smoker), alcohol intake (<20 vs. ≥20 g/day), HEPA (no vs. yes), BMI (<25 vs. ≥25 kg/m^2^), HOMA-IR (<2.5 vs. ≥2.5), hsCRP (<1.0 vs. ≥1.0), diabetes (no vs. yes), hypertension (no vs. yes), and Framingham Risk Score (<10% vs. ≥10%). Interactions between subgroups were tested using likelihood ratio tests comparing models with and without multiplicative interaction terms. All P-values were two-tailed, and P-values < 0.05 were considered statistically significant. We used STATA version 14.0 (Stata Corp., College Station, TX, USA) for data analysis.

### Data availability

All data generated or analysed during this study are included in this published article and its Supplementary Information files.

## Results

### Population characteristics

Table 1 shows the baseline characteristics of our study population according to the presence of NSSTTA. The mean age and proportion of males were 40.2 years (SD: 8.0 years) and 71.1%, respectively. Participants with NSSTTA were more likely to be older and to have a history of hypertension or diabetes compared with those without NSSTTA. Body mass index, systolic and diastolic BP, and levels of fasting glucose, total cholesterol, LDL-C, and HDL-C were higher in the NSSTTA group.

**Table 1:** Baseline characteristics of the study population according to the presence of NSSTTA.

Characteristics	Overall	Without NSSTTA	With NSSTTA	P-value
Number of participants	74,976	73,837	1,139	
Age (years)	40.2 (8.0)	40.2 (7.9)	43.7 (9.8)	<0.001
Male (%)	71.1	71.2	63.5	<0.001
Current smoker (%)	24.6	24.5	26.8	0.093
Alcohol intake^a^ (%)	25.3	25.3	26.5	0.390
Vigorous exercise^b^ (%)	13.1	13.1	14.9	0.082
Education level^c^ (%)	83.2	83.4	76.2	<0.001
Family history of CVD	12.1	12.1	14.8	0.005
History of diabetes	3.1	3.0	4.4	0.008
History of hypertension	10.5	10.3	20.5	<0.001
BMI (kg/m^2^)	23.9 (3.3)	23.9 (3.3)	24.4 (3.6)	<0.001
Systolic BP (mmHg)	110.8 (12.8)	110.7 (12.8)	116.0 (15.7)	<0.001
Diastolic BP (mmHg)	72.0 (10.2)	71.9 (10.2)	74.8 (11.9)	<0.001
Glucose (mg/dl)	96.4 (15.4)	96.4 (15.3)	98.6 (19.6)	<0.001
Uric acid (mg/dl)	5.6 (1.5)	5.6 (1.5)	5.2 (1.5)	0.057
Total cholesterol (mg/dl)	197.6 (34.6)	197.5 (34.6)	201.1 (36.7)	0.001
LDL-C (mg/dl)	123.7 (31.8)	123.6 (31.8)	126.1 (34.1)	0.010
HDL-C (mg/dl)	55.9 (14.5)	55.9 (14.5)	56.9 (15.5)	0.029
Triglycerides (mg/dl)	104 (72–154)	104 (72–154)	105 (72–159)	0.378
ALT (U/L)	20 (14–30)	20 (14–30)	20 (14–31)	0.110
GGT (U/L)	24 (15–41)	24 (15–41)	25 (16–45)	0.010
hsCRP (mg/L)	0.5 (0.3–1.0)	0.5 (0.3–1.0)	0.5 (0.3–1.2)	<0.001
HOMA-IR	1.31 (0.87–1.96)	1.31 (0.87–1.96)	1.31 (0.81–2.03)	0.472

Data are mean (standard deviation), median (interquartile range), or percentage.

ALT, alanine aminotransferase; BMI, body mass index; BP, blood pressure; HDL-C, high-density lipoprotein-cholesterol; hsCRP, high sensitivity C-reactive protein; HOMA-IR, homeostasis model assessment of insulin resistance; LDL-C, low-density lipoprotein-cholesterol.

^a^≥20 g of ethanol per day.

^b^≥3 times per week.

^c^≥College graduate.

### Echocardiography

Table 2 presents echocardiographic parameters. The presence of NSSTTA was associated with lower septal E′, higher E/E′, higher LVEDD, higher LV mass as indexed by height or body surface area, and higher LA dimension compared to those of control individuals.

**Table 2:** Estimated^a^ mean values (95% CIs) of echocardiographic characteristics of the study participants according to the presence of NSSTTA.

Characteristics	Without NSSTTA	With NSSTTA	P-value
Heart rate (bpm)	64.8 (64.7–64.8)	64.2 (63.6–64.7)	0.026
Ejection fraction (%)	66.7 (66.6–66.7)	67.4 (67.1–67.7)	<0.001
E (cm/s)	71.1 (70.8–71.5)	74.8 (72.1–77.4)	0.007
A (cm/s)	53.7 (52.7–54.8)	53.8 (45.4–62.2)	0.989
E/A ratio	1.42 (1.41–1.43)	1.45 (1.41–1.50)	<0.001
E′ (cm/s)	10.6 (10.4–10.8)	9.6 (7.7–11.4)	<0.001
A′ (cm/s)	8.7 (8.5–8.9)	8.6 (7.0–10.1)	0.025
E/E'	7.5 (7.4–7.5)	8.7 (8.3–9.1)	<0.001
LVEDD (mm)	48.8 (48.8–48.9)	49.3 (49.1–49.6)	<0.001
LV mass (g)	133.8 (133.6–134.0)	147.0 (145.4–148.6)	<0.001
LVMI (g/ht2.7, g/m2.7)	32.0 (32.0–32.1)	35.6 (35.2–36.0)	<0.001
LVMI (g/BSA, g/m^2^)	73.9 (73.8–74.0)	80.7 (80.0–81.5)	<0.001
LA dimension (mm)	34.1 (33.9–34.2)	35.1 (34.0–36.2)	<0.001

^a^Adjusted for age and sex.

BSA, body surface area; CI, confidence interval; LA, left atrial; LV, left ventricular; LVEDD, left ventricular end-diastolic diameter; LVMI, left ventricular mass index.

Of the 74,976 subjects with echocardiographic data, 21,118 had impaired LV relaxation and 1,687 had LVH (Table 3). The presence of NSSTTA was associated with an increased prevalence of impaired LV relaxation. In an age- and sex-adjusted model, the odds ratio (95% CI) for impaired LV relaxation comparing participants with NSSTTA to those without was 1.97 (1.72–2.26). After adjustment for age, sex, year of screening exam, smoking status, alcohol intake, physical activity, education level, history of diabetes, history of hypertension, BMI and family history of heart disease, multivariable-adjusted odds ratio (95% CI) for impaired LV relaxation comparing participants with NSSTTA to those without was 1.75 (1.51–2.03) (Table 3, model 1). After further adjustment for metabolic parameters including glucose, LDL-C, HDL-C, triglycerides and systolic blood pressure (Table 3, model 2), the association was slightly attenuated but remained significant with corresponding odds ratio (95% CI) of 1.55 (1.33–1.80). We also examined the association between the presence of NSSTTA and echocardiographic LVH. After adjustment for age, sex, year of screening exam, smoking status, alcohol intake, physical activity, education level, history of diabetes, history of hypertension, BMI and family history of heart disease, the multivariable-adjusted odds ratio (95% CI) for echocardiographic LVH comparing individuals with NSSTTA to those without was 3.58 (2.86–4.49) (Table 3, model 1). After further adjustment for metabolic parameters and systolic blood pressure, corresponding odds ratio (95% CI) for echocardiographic LVH was 3.15 (2.51–3.96).

**Table 3:** Odds ratios (95% CIs) of impaired LV relaxation and LV hypertrophy according to the presence of NSSTTA.

	Number	Cases	Age- and sex-adjusted OR^a^ (95% CI)	Multivariate-adjusted OR^a^	
				Model 1	Model 2
**Impaired LV relaxation**					
Without NSSTTA	73,837	20,577	1.00 (reference)	1.00 (reference)	1.00 (reference)
With NSSTTA	1,139	541	1.97 (1.72–2.26)	1.75 (1.51–2.03)	1.55 (1.33–1.80)
**LV hypertrophy**					
Without NSSTTA	73,837	1,552	1.00 (reference)	1.00 (reference)	1.00 (reference)
With NSSTTA	1,139	135	4.15 (3.38–5.09)	3.58 (2.86–4.49)	3.15 (2.51–3.96)

^a^Estimated from logistic regression models. Multivariable model 1 was adjusted for age, sex, center, year of screening exam, smoking status, alcohol intake, physical activity, educational level, family history of heart disease, history of diabetes, history of hypertension, and BMI; model 2 was adjusted for the same parameters as model 1, plus levels of glucose, LDL-C, HDL-C, and triglycerides, and systolic blood pressure. Abbreviations are as in Tables 1 and 2.

In pre-specified subgroup analyses, the association between the presence of NSSTTA and impaired LV relaxation was stronger in the intermediate to high CVD-risk group than in the low-risk group according to the Framingham Risk Score stratification (P for interaction = 0.02) (Table 4); otherwise, there were no significant interactions by other pre-specified subgroups including sex (male vs. female), age (<50 vs. ≥50 years), current smoking (no vs. yes), alcohol intake (<20 vs. ≥20 g/day), HEPA (no vs. yes), BMI (<25 vs. ≥25 kg/m^2^), HOMA-IR (<2.5 vs. ≥2.5), hsCRP (<1.0 vs. ≥1.0), diabetes (no vs. yes), and hypertension (no vs. yes) (Table 4). The associations between NSSTTA and echocardiographic LVH were similar across all pre-specified subgroups without no significant interaction (Appendix Table 1).

**Table 4:** Odds ratios^a^ (95% CIs) of impaired LV relaxation according to the presence of NSSTTA in clinically relevant subgroups.

Subgroup	Without NSSTTA	With NSSTTA	P for interaction
Sex			0.36
Female (n = 21,694)	1.00 (reference)	1.75 (1.31–2.36)	
Male (m = 53,282)	1.00 (reference)	1.51 (1.26–1.80)	
Age			0.62
<50 years (n = 66,976)	1.00 (reference)	1.74 (1.48–2.04)	
≥50 years (n = 8,000)	1.00 (reference)	1.62 (1.14–2.32)	
Current smoker			0.18
No (n = 51,677)	1.00 (reference)	1.40 (1.16–1.70)	
Yes (n = 16,818)	1.00 (reference)	1.84 (1.38–2.45)	
Alcohol intake			0.90
<20 g/day (n = 52,816)	1.00 (reference)	1.53 (1.27–1.85)	
≥20 g/day (n = 17,930)	1.00 (reference)	1.60 (1.21–2.13)	
HEPA			0.14
No (n = 62,063)	1.00 (reference)	1.66 (1.40–1.97)	
Yes (n = 11,253)	1.00 (reference)	1.22 (0.87–1.72)	
BMI			0.46
<25 kg/m^2^ (n = 49,261)	1.00 (reference)	1.65 (1.35–2.02)	
≥25 kg/m^2^ (n = 25,715)	1.00 (reference)	1.48 (1.18–1.86)	
HOMA-IR			0.08
<2.5 (n = 64,161)	1.00 (reference)	1.63 (1.37–1.92)	
≥2.5 (n = 10,707)	1.00 (reference)	1.29 (0.91–1.83)	
hsCRP			0.79
<1.0 mg/l (n = 53,493)	1.00 (reference)	1.52 (1.26–1.84)	
≥1.0 mg/l (n = 18,283)	1.00 (reference)	1.61 (1.21–2.13)	
Diabetes			0.57
No (n = 71,283)	1.00 (reference)	1.56 (1.33–1.83)	
Yes (n = 3,693)	1.00 (reference)	1.40 (0.80–2.45)	
Hypertension			0.91
No (n = 64,570)	1.00 (reference)	1.56 (1.31–1.86)	
Yes (n = 10,406)	1.00 (reference)	1.52 (1.14–2.04)	
Framingham Risk Score			0.02
<10% (n = 61,651)	1.00 (reference)	1.39 (1.17–1.66)	
≥10% (n = 6,844)	1.00 (reference)	2.46 (1.61–3.75)	

^a^Estimated from logistic regression models. Multivariable model was adjusted for age, sex, center, year of screening exam, smoking status, alcohol intake, physical activity, educational level, BMI, family history of heart disease, history of diabetes, history of hypertension, levels of glucose, LDL-C, HDL-C, and triglycerides, and systolic blood pressure.

## Discussion

In this large study of young and middle-aged men and women, we identified two major findings. First, we found an association between the presence of NSSTTA on ECG and impaired LV relaxation and LVH on echocardiography. These associations persisted even after adjustment for potential confounders, including cardiovascular risk factors. Second, the association between NSSTTA and impaired LV relaxation was stronger in the intermediate to high CVD-risk group than in the low-risk group according to Framingham Risk Score stratification.

Previous studies have examined the associations between ECG findings and diastolic function of the LV^16–20^. The P-wave dispersion, QRS-voltage-duration products, QT interval, T-wave inversion, and T-peak to T-end interval are ECG findings or measurements observed at various points in the cardiac cycle that have been found to be related to diastolic dysfunction^16–20^. Several of these studies, and ours, present data that supports that the main mechanism explaining the relationship between LV diastolic dysfunction and ECG abnormalities is related to abnormal repolarization of electrical activity^17,18^. The difference between the ECG findings in our study from those of previous studies is that NSSTTA are commonly assessed in clinical settings, whereas previous study parameters are not easily applicable in clinical practice^17,18^.

The mechanisms underlying the association between NSSTTA with impaired LV relaxation and echocardiographic LVH are not fully understood. We demonstrated a higher prevalence of echocardiographic LVH in the context of NSSTTA without ECG LVH. Most of the ECG LVH criteria include high electrical voltage with the assumption that thicker myocardium has more electrical activity. Indeed, the ECG characteristics of physiological LVH include QRS high voltage^21^, whereas pathological LVH might not present with a QRS high voltage pattern. Pathological LVH is accompanied by not only increases in levels of certain cardiomyocyte proteins, but also an altered extracellular matrix composed of fibroblasts, cardiac steatosis, and vascular smooth muscle cells^22^. This can explain the discrepancy between anatomical LV mass and electrically active LV mass. In a report of LVH patients without coronary stenosis, the electrocardiographic findings of echocardiographic LVH presented as various electrical manifestations, including a flat T wave without increased QRS voltage^23^. Indeed, it has recently been shown that myocardial fibrosis measured by cardiovascular magnetic resonance imaging (MRI) is associated with reduced QRS voltage regardless of LV mass on ECG^24^.

In the present study, the association between NSSTTA and impaired LV relaxation was stronger in individuals at an intermediate to high risk for CVD. A previous community-based study demonstrated that a higher prevalence of diastolic dysfunction was observed as the number of cardiovascular risk factors increased^25^. The reasons for the stronger association of NSSTTA with impaired LV relaxation in this patient subgroup are unclear, but cardiac fibrosis as a cause of diastolic dysfunction can be more prevalent in the higher risk group, serving as one possible explanation^26^.

Cardiac fibrosis is responsible for electrical disturbances that can lead to ST-T wave abnormalities. Increased myocardial interstitial fibrosis and collagen accumulation commonly accompany LVH, which affects conduction disturbances, myofibrillar disarray, and heterogeneous gap junction distribution^27^. Lindsay *et al*. reported a significant elevation in level of TIMP-1, a biomarker of cardiac fibrosis, in patients with hypertension and ECG ST-T changes^28^. In addition, level of TIMP-1 was related to the E/A ratio and DT on echocardiography, indicating the possibility of a correlation between the expression of this protein with LV diastolic function^28^. Moreo *et al*. reported an association between cardiac fibrosis and diastolic dysfunction using cardiac MRI and echocardiography^29^.

Our study had some limitations. First, we measured mitral annulus velocity only in the septal area. The current guideline recommends the use of a mean value by measuring both septal and lateral mitral annulus velocities; however, septal E′ can predict LV longitudinal myocardial relaxation and diastolic function^30^. Second, other ECG parameters related to LV diastolic dysfunction reported in previous studies were not available in our study. However, our study was the first to find an association between NSSTTA and impaired LV relaxation, explaining the poor cardiovascular prognosis of NSSTTA from prior studies. Our findings in apparently healthy, young, and middle-aged Korean adults might limit generalizability to other ethnic populations or patients with comorbid conditions. However, subjects with fewer comorbidities minimize the presence of potentially unmeasured confounders that could affect ECG signals or LV diastolic function.

## Conclusion

In this large sample of apparently healthy Korean adults, NSSTTA were associated with increased prevalence of impaired LV relaxation and LVH on echocardiography. In particular, clinicians should be mindful of impaired LV relaxation in the presence of NSSTTA on ECG when the conventional cardiovascular risk is intermediate to high. This suggests that NSSTTA can reflect subclinical cardiac dysfunction and geometric abnormalities, possibly explaining the unfavorable prognosis of NSSTTA with regard to cardiovascular outcomes.

## Electronic supplementary material

Supplementary Information
