# Supplementary material for: Association of isolated minor nonspecific ST-T abnormalities with left ventricular hypertrophy and diastolic dysfunction
Source: Sci Rep. 2018 Jun 8;8:8791. doi: 10.1038/s41598-018-27028-6 (PMC5993779; doi:10.1038/s41598-018-27028-6)
Supplement: Supplementary file 1 — Supplementary Information [file 41598_2018_27028_MOESM1_ESM.docx]

**Association of isolated minor nonspecific ST-T abnormalities with left ventricular hypertrophy and diastolic dysfunction**

Jeong Gyu Kang^a^, Yoosoo Chang^a,b,c^, Ki-Chul Sung^d^, Jang-Young Kim^e^, Hocheol Shin^f^, Seungho Ryu^a,b,c^

^a^ Center for Cohort Studies, Total Healthcare Center, Kangbuk Samsung Hospital, Sungkyunkwan University, School of Medicine, Seoul, South Korea

^b^ Department of Occupational and Environmental Medicine, Kangbuk Samsung Hospital, Sungkyunkwan University, School of Medicine, Seoul, South Korea

^c^ Department of Clinical Research Design & Evaluation, SAIHST, Sungkyunkwan University, Seoul, South Korea

^d^ Division of Cardiology, Department of Internal Medicine, Kangbuk Samsung Hospital, Sungkyunkwan University School of Medicine, Seoul, South Korea

^e^ Departments of Cardiology, Wonju College of Medicine, Yonsei University, Wonju, South Korea; Institute of Genomic Cohort, Yonsei University,Wonju, South Korea

^f^ Department of Family Medicine, Kangbuk Samsung Hospital, Sungkyunkwan University School of Medicine, Seoul, South Korea

**Appendix table 1. Odds ratios^a^ (95% CIs) of left ventricular hypertrophy according to the presence of NSSTTA in clinically relevant subgroups**

| Subgroup | Without NSSTTA | With NSSTTA | P for interaction |
| --- | --- | --- | --- |
| Sex |  |  | 0.09 |
| Female (n=21,694) | 1.00 (reference) | 2.52 (1.77-3.58) |  |
| Male (n=53,282) | 1.00 (reference) | 3.70 (2.76-4.96) |  |
| Age |  |  | 0.34 |
| <50 years (n=66,976) | 1.00 (reference) | 3.59 (2.68-4.82) |  |
| ≥50 years (n=8,000) | 1.00 (reference) | 2.94 (2.08-4.15) |  |
| Current smoker |  |  | 0.79 |
| No (n=51,677) | 1.00 (reference) | 3.18 (2.36-4.28) |  |
| Yes (n=16,818) | 1.00 (reference) | 3.37 (2.16-5.26) |  |
| Alcohol intake |  |  | 0.43 |
| <20 g/day (n=52,816) | 1.00 (reference) | 3.10 (2.31-4.14) |  |
| ≥20 g/day (n=17,930) | 1.00 (reference) | 3.81 (2.48-5.87) |  |
| HEPA |  |  | 0.26 |
| No (n=62,063) | 1.00 (reference) | 3.38 (2.61-4.36) |  |
| Yest (n=11,253) | 1.00 (reference) | 2.48 (1.51-4.08) |  |
| BMI |  |  | 0.39 |
| <25kg/m^2^ (n=49,261) | 1.00 (reference) | 3.46 (2.36-5.08) |  |
| ≥25kg/m^2^ (n=25,715) | 1.00 (reference) | 2.99 (2.27-3.95) |  |
| HOMA-IR |  |  | 0.29 |
| <2.5 (n= 64,161) | 1.00 (reference) | 2.91 (2.22-3.82) |  |
| ≥2.5 (n=10,707) | 1.00 (reference) | 3.75 (2.43-5.78) |  |
| hsCRP |  |  | 0.36 |
| <1.0 mg/l (n= 53,493) | 1.00 (reference) | 3.03 (2.18-4.21) |  |
| ≥1.0 mg/l (n=18,283) | 1.00 (reference) | 3.48 (2.44-4.98) |  |
| Diabetes |  |  | 0.44 |
| No (n=71,283) | 1.00 (reference) | 3.05 (2.38-3.91) |  |
| Yes (n=3,693) | 1.00 (reference) | 3.69 (2.08-6.56) |  |
| Hypertension |  |  | 0.34 |
| No (n=64,570) | 1.00 (reference) | 2.69 (1.91-3.79) |  |
| Yes (n=10,406) | 1.00 (reference) | 3.46 (2.55-4.70) |  |
| Framingham Risk Score |  |  | 0.30 |
| <10% (n=61,651) | 1.00 (reference) | 2.89 (2.11-3.96) |  |
| ≥10% (n=6,844) | 1.00 (reference) | 3.76 (2.52-5.62) |  |

^a^ Estimated from logistic regression models. Multivariable model was adjusted for age, sex, center, year of screening exam, smoking status, alcohol intake, physical activity, educational level, BMI, family history of heart disease, history of diabetes, history of hypertension, levels of glucose, LDL-C, HDL-C, and triglycerides, and systolic blood pressure
